# Performance of a machine-learning algorithm to predict hypotension in mechanically ventilated patients with COVID-19 admitted to the intensive care unit: a cohort study

**DOI:** 10.1007/s10877-021-00778-x

**Published:** 2021-11-13

**Authors:** Ward H. van der Ven, Lotte E. Terwindt, Nurseda Risvanoglu, Evy L. K. Ie, Marije Wijnberge, Denise P. Veelo, Bart F. Geerts, Alexander P. J. Vlaar, Björn J. P. van der Ster

**Affiliations:** 1 Department of Anesthesiology, Amsterdam UMC, University of Amsterdam, Meibergdreef 9, Amsterdam, The Netherlands; 2 Department of Intensive Care, Spaarne Gasthuis, Boerhaavelaan 22, Haarlem, The Netherlands; 3 Department of Intensive Care, Amsterdam UMC, University of Amsterdam, Meibergdreef 9, Amsterdam, The Netherlands; 4 Laboratory of Experimental Intensive Care and Anesthesiology, Amsterdam UMC, University of Amsterdam, Meibergdreef 9, Amsterdam, The Netherlands

**Keywords:** COVID-19, Hemodynamics, Hypotension, Intensive care unit, Machine-learning, Validation

## Abstract

The Hypotension Prediction Index (HPI) is a commercially available machine-learning algorithm that provides warnings for impending hypotension, based on real-time arterial waveform analysis. The HPI was developed with arterial waveform data of surgical and intensive care unit (ICU) patients, but has never been externally validated in the latter group. In this study, we evaluated diagnostic ability of the HPI with invasively collected arterial blood pressure data in 41 patients with COVID-19 admitted to the ICU for mechanical ventilation. Predictive ability was evaluated at HPI thresholds from 0 to 100, at incremental intervals of 5. After exceeding the studied threshold, the next 20 min were screened for positive (mean arterial pressure (MAP) < 65 mmHg for at least 1 min) or negative (absence of MAP < 65 mmHg for at least 1 min) events. Subsequently, sensitivity, specificity, positive predictive value (PPV), negative predictive value (NPV), and time to event were determined for every threshold. Almost all patients (93%) experienced at least one hypotensive event. Median number of events was 21 [7–54] and time spent in hypotension was 114 min [20–303]. The optimal threshold was 90, with a sensitivity of 0.91 (95% confidence interval 0.81–0.98), specificity of 0.87 (0.81–0.92), PPV of 0.69 (0.61–0.77), NPV of 0.99 (0.97–1.00), and median time to event of 3.93 min (3.72–4.15). Discrimination ability of the HPI was excellent, with an area under the curve of 0.95 (0.93–0.97). This validation study shows that the HPI correctly predicts hypotension in mechanically ventilated COVID-19 patients in the ICU, and provides a basis for future studies to assess whether hypotension can be reduced in ICU patients using this algorithm.

## Introduction

During the first wave of coronavirus disease 2019 (COVID-19) in the spring of 2020, approximately 5% of infected patients with symptoms and up to 24% of hospitalized patients were admitted to the intensive care unit (ICU) due to respiratory failure requiring invasive ventilation [1–6]. Since COVID-19 is primarily a pulmonary disease, attention has mainly been drawn to optimal ventilation techniques. However, many patients also experience circulatory failure, reflected by the majority requiring vasopressors to correct arterial hypotension [6–9].

Hypotension in general ICU patients is associated with acute kidney injury (AKI), myocardial injury, and mortality [10–14]. While similar research is not yet available for COVID-19 patients admitted to the ICU, the disease can manifest with myocardial infarction, heart failure, and cardiogenic shock [15], which could directly influence organ perfusion. The Surviving Sepsis Campaign recommends maintaining mean arterial pressure (MAP) in COVID-19 patients admitted to the ICU at 60–65 mmHg, to preserve adequate tissue perfusion [16]. This can, however, be challenging in rapidly changing respiratory and circulatory conditions, and hypotension in COVID-19 patients may therefore not always be prevented during routine care.

Prevention of hypotension may be realized using predictive algorithms, providing early warnings for impending events, which offers the opportunity for intervention before the actual event occurs. In 2018, the Hypotension Prediction Index (HPI) was presented, a predictive algorithm created with machine-learning. The algorithm is trained on invasive arterial waveform data of 1334 patients that were admitted to the ICU or who underwent surgery [17]. Predictions of the HPI are based on arterial waveform changes over time, and are displayed as a continuously changing number between 0 and 100, reflecting the probability of impending hypotension. According to the developers, hypotensive events were accurately predicted 5–15 min in advance, with sensitivity and specificity of 0.87–0.92 [17].

Although the HPI was developed using data from ICU patients too, external and clinical validation has, thus far, only been conducted in surgical patients [17]. Therefore, performance of the HPI remains unknown for general ICU patients, and COVID-19 patients in particular. This study assesses the diagnostic ability of the HPI applied to invasively collected arterial blood pressure data of COVID-19 patients admitted to the ICU for mechanical ventilation.

## Methods

This was a single-center, prospective, observational study in COVID-19 patients admitted to the ICU for mechanical ventilation at the Amsterdam UMC, location Academic Medical Center (AMC). Data were collected from the 10th of April 2020 until the 29th of May 2020. The study was approved by the Ethics Committee of the Amsterdam UMC, location AMC (Date: April 8th, 2020/No. W20_160#20.181), prior to data collection. All patients surviving their ICU stay or their legal representatives provided written consent. Patients were eligible if they had a confirmed infection with severe acute respiratory syndrome coronavirus 2, had an arterial catheter in the radial artery and were expected to receive mechanical ventilation for at least eight h onwards. To obtain sufficient data for validation, we intended to collect data continuously for three consecutive days in all patients. The arterial catheter was connected to an Acumen IQ pressure transducer (Edwards Lifesciences, Irvine, CA, USA), placed at the level of the right atrium and zeroed before start of measurements and every eight h afterwards. Fast flush tests were performed to ensure that the system was not over- or underdamped [18]. The transducer measured arterial blood pressure and derived advanced hemodynamic parameters from the arterial waveform every 20 s, which were subsequently displayed on EV1000 or HemoSphere monitors (Edwards Lifesciences, Irvine, CA, USA). The monitors were blinded for treating ICU personnel throughout the measurement period. Interventions were at the discretion of the treating intensivist and were not influenced by this study. After measurements were finished, data were downloaded and HPI values were calculated at 20-s intervals post hoc. Study conduct and reporting were done in accordance with the STROBE and STARD guidelines [19, 20].

### Statistical analysis

Normally distributed data are presented as mean with standard deviation (SD), non-normally distributed data as median [25th–75th percentiles], and categorical data as n (%). Hypotension was defined as a MAP < 65 mmHg for at least 1 min. For each patient, monitoring time, number of hypotensive events, time spent in hypotension, area under the threshold and the time-weighted average (TWA) of hypotension were determined. The TWA of hypotension is a function that incorporates both hypotension duration and severity, corrected for the total monitoring time [21].

The predictive ability of the HPI was determined using a forward analysis similar to Wijnberge et al. [22], starting with an HPI value exceeding the studied threshold for at least 1 min, after which the next 20 min following the crossing of the threshold were screened for positive (hypotensive) events. Positive events were defined as a MAP < 65 mmHg for at least 1 min. If the HPI remained below the studied threshold for more than 1 min, the next 20-min timeframe was screened for negative (non-hypotensive) events, which were defined as the absence of a MAP < 65 mmHg for at least 1 min. Consequently, timeframes were labeled as true positive (TP), false positive (FP), true negative (TN), or false negative (FN). After TP, FP, or FN timeframes, the window was shifted 20 min forward in time, to avoid that events would be counted multiple times. To correct for an overrepresentation of TNs, these timeframes were counted with a maximum of one event every 20 min. Events were considered to have ended when in the first 20-s sample following the event, the MAP reached a value of at least 65 mmHg again.

We excluded measurements that were most likely influenced by clinical interventions, such as administration of vasopressors (mainly norepinephrine in our institution) or application of positional changes (for instance Trendelenburg position), resulting in a rapid rise in blood pressure. These measurements would otherwise have been classified as false positives, unjustly reducing the predictive ability of the HPI. Therefore, data with an increase in MAP of ≥ 5 mmHg within 20 s or an increase in MAP of ≥ 8 mmHg within 2 min, starting from a baseline MAP < 70 mmHg, were censored, in accordance with earlier studies [17, 22–24].

From the TPs, FPs, TNs, and FNs, sensitivity ($\frac{\#\mathrm{TPs}}{(\#\mathrm{TPs}+\#\mathrm{FNs})}$), specificity ($\frac{\#\mathrm{TNs}}{(\#\mathrm{TNs}+\#\mathrm{FPs})}$), positive predictive value (PPV; $\frac{\#\mathrm{TPs}}{(\#\mathrm{TPs}+\#\mathrm{FPs})}$), and negative predictive value (NPV; $\frac{\#\mathrm{TNs}}{(\#\mathrm{TNs}+\#\mathrm{FNs})}$) were calculated at every HPI threshold from 0 to 100 with incremental intervals of 5. Youden’s J statistic was calculated to determine the optimal HPI threshold [25]. A receiver operating characteristic (ROC) curve was plotted and the area under the curve (AUC) was calculated. Furthermore, median time-to-event and event rate were determined for every HPI threshold. The event rate is computed as the number of hypotensive events that follow an HPI alarm. To compensate for repeated measurements in this relatively small sample size, a bootstrapping procedure was performed with 100,000 repetitions, resulting in bootstrap-corrected sample estimates with a 95% confidence interval (CI) for sensitivity, specificity, PPV, NPV, time to event, and event rate. Statistical analyses were performed with RStudio (R Foundation for Statistical Computing, Vienna, Austria) and MATLAB (MathWorks, Natick, MA, USA). Graphs were made with GraphPad Prism (GraphPad Software, San Diego, CA, USA).

## Results

Data were collected in 41 patients whose characteristics are reported in Table 1. Mean age was 60 (9) years and 61% were male. Median BMI was 28 [26–31], 73% of the patients had underlying diseases, primarily chronic arterial hypertension and diabetes. Median ICU length of stay was 18 [11–25] days and 44% of the patients died in the ICU. More than half of the population developed AKI, and 20% required renal replacement therapy. The majority (71%) experienced thrombotic events, such as venous thromboembolism (54%) and pulmonary embolism (44%).

**Table 1:** Baseline characteristics

Variable	Value (n = 41)
Age, (yr) Min–max	60 (9) 34–76
Male sex	25 (61)
Weight, (kg)	82 [76–94]
Height, (cm)	173 (10)
BMI, (kg·m^−1^)	28 [26–31]
Current smoker	7 (17)
*Comorbidities*	
Chronic arterial hypertension	20 (49)
Diabetes (type 1 and 2)	13 (32)
Stroke	3 (7)
COPD	3 (7)
Asthma	3 (7)
Myocardial infarction	2 (5)
CKD	1 (2)
*Home medication*	
Antidiabetic	12 (29)
Cholesterol lowering drug	11 (27)
ACE inhibitor	8 (20)
Beta blocker	8 (20)
Calcium channel blocker	8 (20)
Diuretic	7 (17)
Angiotensin II receptor blocker	4 (10)
Platelet inhibitor	6 (15)
Anticoagulant	2 (5)
Symptom duration before hospital admission, (d)	7 [6–10]
*Complications during ICU stay*	
AKI	23 (56)
AKI requiring RRT	8 (20)
DVT, confirmed with US	22 (54)
PE, confirmed with CT	18 (44)
Tracheostomy	9 (22)
Deceased	18 (44)
ICU LOS, (d)	18 [11–25]
In-hospital LOS, (d)	26 [15–45]

Data presented as number (%), mean (standard deviation) or median [25th–75th percentiles]. Antidiabetic includes both oral antidiabetics and insulin

*BMI* body mass index, *COPD* chronic obstructive pulmonary disease, *CKD* chronic kidney disease, *ACE* angiotensin-converting enzyme, *ICU* intensive care unit, *AKI* acute kidney injury, *RRT* renal replacement therapy, *DVT* deep vein thrombosis, *US* ultrasound, *PE* pulmonary embolism, *CT* computed tomography, *LOS* length of stay

Hypotension statistics of the patients are reported in Table 2. Cumulative monitoring time of all patients was 2822 h (118 days). Median monitoring time per patient was 70 [45–77] h. Almost all patients (93%) experienced at least one hypotensive event. In total, 1,454 hypotensive events were recorded. Median number of hypotensive events per patient was 21 [7–54] and ranged from 0 to 177. Median time spent in hypotension was 114 min [20–303], which translates to 3% [1–9] of case time. The area under the threshold (MAP of 65 mmHg) per patient was 286.44 mmHg·min [41.52–998.42]. Median TWA of hypotension was 0.08 mmHg [0.01–0.26].

**Table 2:** Hypotension statistics

Variable	Value
Monitoring time of all patients combined, (h)/(min)	2,822/169,342
Monitoring time per patient, (h)/(min)	70 [45–77]/4223 [2723–4615]
Patients with hypotension	38 (93)
Total number of hypotensive events	1,454
Hypotensive events per patient Min–max	21 [7–54] 0–177
Total hypotension duration of all patients combined, (min)	8,487
Hypotension duration per patient, (min)	114 [20–303]
Hypotension duration per patient, (% of case time)	3 [1–9]
AUT MAP 65 mmHg per patient, (mmHg**·**min)	286.44 [41.52–998.42]
TWA of hypotension per patient, (mmHg)	0.08 [0.01–0.26]

Data presented as number (%) or median [25th–75th percentiles]. Hypotension is defined a as MAP < 65 mmHg for at least 1 min

*AUT* area under the threshold, *MAP* mean arterial pressure, *TWA* time-weighted average

**Fig. 1:**
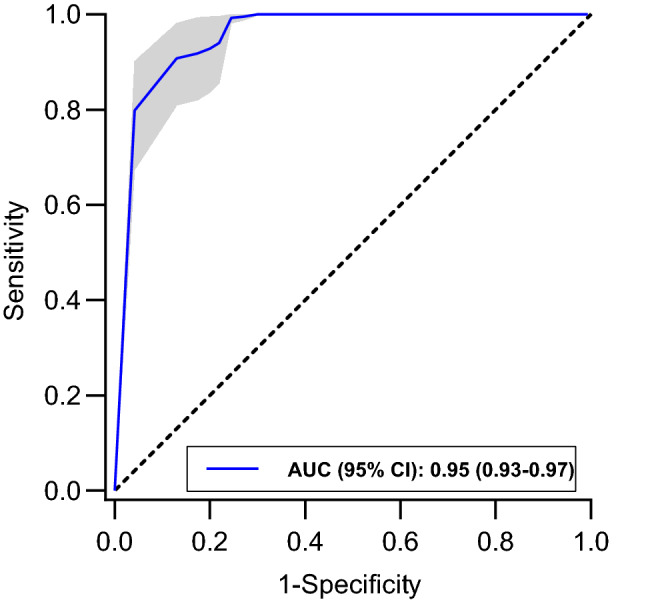
Receiver operating characteristic curve with 95% CI of the HPI for prediction of hypotensive events. Hypotension is defined as a MAP < 65 mmHg for at least 1 min. Dashed line represents the line of identity, where sensitivity equals 1-specificity. *AUC* area under the curve, *CI* confidence interval, *HPI* Hypotension Prediction Index, *MAP* mean arterial pressure

**Fig. 2:**
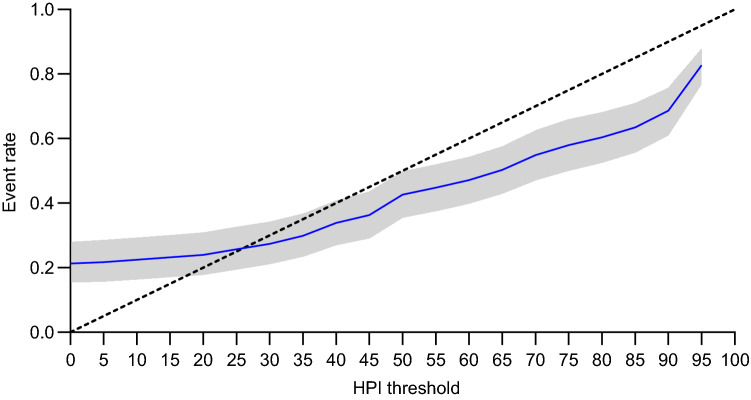
Event rate with 95% CI in relation to incremental HPI thresholds. Dashed line represents the line of identity. *CI* confidence interval, *HPI* Hypotension Prediction Index

Table 3 lists sensitivity, specificity, Youden’s J statistic, PPV, NPV, time to event, and event rate for HPI thresholds between 0 and 100 at incremental intervals of 5. While sensitivity and NPV remained equal (1.00) from thresholds 0 up to 60, specificity and PPV showed a gradual increase (0.01 to 0.70 and 0.30 to 0.55, respectively). Median time to event gradually decreased from lower towards higher HPI thresholds. The HPI threshold of 80 yielded a sensitivity of 0.93 (95% CI 0.84–0.99), specificity of 0.80 (0.72–0.87), PPV of 0.62 (0.53–0.71), and NPV of 0.99 (0.98–1.00). The HPI threshold of 85, which is the threshold used in the majority of clinical trials, performed better with a sensitivity of 0.92 (0.82–0.99), specificity of 0.83 (0.76–0.89), PPV of 0.64 (0.55–0.72), and NPV of 0.99 (0.97–1.00). The optimal HPI threshold was at 90 (Youden’s J statistic of 0.78), demonstrating a sensitivity of 0.91 (0.81–0.98), specificity of 0.87 (0.81–0.92), PPV of 0.69 (0.61–0.77), NPV of 0.99 (0.97–1.00) and median time to event of 3.93 min (3.72–4.15). Figure 1 shows the ROC curve with an AUC of 0.95 (0.93–0.97). Event rate for incremental HPI thresholds is presented in Fig. 2.

**Table 3:** Bootstrap-corrected discriminative ability, Youden’s J statistic, time to event, and event rate at incremental HPI thresholds

HPI threshold	Sensitivity	Specificity	Youden’s J statistic	PPV	NPV	Time to event, (min)	Event rate
0	1.00 (1.00–1.00)	0.01 (0.00–0.02)	0.01	0.30 (0.22–0.38)	1.00 (1.00–1.00)	4.96 (4.74–5.17)	0.21 (0.15–0.28)
5	1.00 (1.00–1.00)	0.12 (0.06–0.19)	0.12	0.30 (0.21–0.38)	1.00 (1.00–1.00)	4.85 (4.64–5.07)	0.22 (0.16–0.29)
10	1.00 (1.00–1.00)	0.20 (0.12–0.28)	0.20	0.31 (0.28–0.40)	1.00 (1.00–1.00)	4.76 (4.55–4.97)	0.22 (0.16–0.29)
15	1.00 (1.00–1.00)	0.26 (0.17–0.35)	0.26	0.33 (0.24–0.42)	1.00 (1.00–1.00)	4.74 (4.53–4.96)	0.23 (0.17–0.30)
20	1.00 (1.00–1.00)	0.31 (0.22–0.41)	0.31	0.33 (0.25–0.43)	1.00 (1.00–1.00)	4.69 (4.48–4.90)	0.24 (0.18–0.31)
25	1.00 (1.00–1.00)	0.37 (0.27–0.46)	0.37	0.35 (0.26–0.44)	1.00 (1.00–1.00)	4.68 (4.47–4.89)	0.26 (0.19–0.33)
30	1.00 (1.00–1.00)	0.44 (0.34–0.54)	0.44	0.37 (0.28–0.46)	1.00 (1.00–1.00)	4.60 (4.39–4.81)	0.27 (0.21–0.34)
35	1.00 (1.00–1.00)	0.52 (0.42–0.62)	0.52	0.39 (0.30–0.48)	1.00 (1.00–1.00)	4.61 (4.40–4.82)	0.30 (0.23–0.37)
40	1.00 (1.00–1.00)	0.57 (0.47–0.67)	0.57	0.42 (0.34–0.51)	1.00 (1.00–1.00)	4.51 (4.30–4.73)	0.34 (0.27–0.41)
45	1.00 (1.00–1.00)	0.61 (0.51–0.71)	0.61	0.45 (0.36–0.54)	1.00 (1.00–1.00)	4.47 (4.26–4.68)	0.36 (0.29–0.44)
50	1.00 (1.00–1.00)	0.64 (0.54–0.74)	0.64	0.52 (0.43–0.60)	1.00 (1.00–1.00)	4.41 (4.20–4.62)	0.43 (0.36–0.50)
55	1.00 (1.00–1.00)	0.67 (0.57–0.76)	0.67	0.54 (0.45–0.62)	1.00 (1.00–1.00)	4.41 (4.20–4.62)	0.45 (0.38–0.52)
60	1.00 (1.00–1.00)	0.70 (0.60–0.79)	0.70	0.55 (0.46–0.64)	1.00 (1.00–1.00)	4.36 (4.15–4.57)	0.47 (0.40–0.54)
65	0.99 (0.99–1.00)	0.73 (0.64–0.81)	0.72	0.57 (0.48–0.66)	1.00 (0.99–1.00)	4.29 (4.09–4.50)	0.50 (0.43–0.58)
70	0.99 (0.98–1.00)	0.76 (0.67–0.83)	0.75	0.60 (0.51–0.68)	1.00 (0.99–1.00)	4.22 (4.01–4.43)	0.55 (0.47–0.63)
75	0.94 (0.86–1.00)	0.78 (0.70–0.85)	0.72	0.61 (0.52–0.70)	0.99 (0.98–1.00)	4.23 (4.01–4.45)	0.58 (0.50–0.66)
80	0.93 (0.84–0.99)	0.80 (0.72–0.87)	0.73	0.62 (0.53–0.71)	0.99 (0.98–1.00)	4.20 (3.98–4.43)	0.60 (0.53–0.68)
85	0.92 (0.82–0.99)	0.83 (0.76–0.89)	0.75	0.64 (0.55–0.72)	0.99 (0.97–1.00)	4.10 (3.89–4.33)	0.63 (0.56–0.71)
90	0.91 (0.81–0.98)	0.87 (0.81–0.92)	0.78	0.69 (0.61–0.77)	0.98 (0.97–1.00)	3.93 (3.72–4.15)	0.69 (0.61–0.76)
95	0.80 (0.67–0.90)	0.96 (0.94–0.98)	0.76	0.81 (0.73–0.87)	0.98 (0.96–0.99)	3.54 (3.27–3.82)	0.83 (0.77–0.88)
100	0.00 (0.00–0.00)	1.00 (1.00–1.00)	0.00	-	0.80 (0.74–0.85)	-	-

Data presented as median (95% CI)

*CI* confidence interval, *HPI* Hypotension Prediction Index, *PPV* positive predictive value, *NPV* negative predictive value

## Discussion

This validation study shows that the HPI correctly predicts hypotension prior to the actual event, in mechanically ventilated COVID-19 patients in the ICU. Although analyses were performed in a subgroup of ICU patients only, this is the first time that the HPI is externally validated in critically ill patients. Discrimination ability of the HPI algorithm in this cohort can be considered excellent, with an AUC of 0.95.

Notably, for all HPI thresholds between 0 and 90, sensitivity was > 0.9, while specificity steadily increased and reached a value of > 0.8 at HPI thresholds of 80 and above. This implies that the HPI is able to rule out hypotensive events with great certainty. Furthermore, the higher HPI thresholds also demonstrated satisfactory performance at identifying impending hypotension. The optimal threshold at which both sensitivity and specificity are at its maximum, can be mathematically determined with the Youden's J statistic [25], which is a trade-off between the two. In this study, the optimal HPI threshold was 90, with a sensitivity of 0.91 and specificity of 0.87, a Youden’s J statistic of 0.78, and a median time to event of 3.93 min. This threshold differs significantly from previous studies, reporting optimal HPI thresholds between 22 and 41 [17, 23, 24]. A possible explanation is that we applied a different type of analysis, compared to previous trials. As described in detail by Wijnberge et al. [22], the forward analysis also performed in this study, relates better to clinical practice since an increased HPI value is the starting point, rather than the hypotensive event itself. Differences in the analysis could have led us to label certain timeframes differently than we would have in the case of a backward analysis, which presumably yields other results. Furthermore, the vastly different setting of this study and the contrasting hemodynamic profile of the COVID-19 patient, compared to a surgical patient, may have contributed. Although the majority of COVID-19 patients in the ICU receive vasopressors [6–9], they are considered hemodynamically relatively stable [26]. Surgical patients on the other hand, receive frequent interventions and surgical manipulation, resulting in decreased hemodynamic stability. This is supported by the hypotension statistics reported in this study. In our cohort, relatively little hypotension was reported (median 3% of case time, versus 6–11% in surgical patients) [17, 23, 27], which probably results in fewer FN predictions of the HPI, thus leading to a high sensitivity for almost all studied thresholds. The optimal HPI threshold is therefore mainly determined by the reported specificity, which gradually increased towards higher HPI values, due to fewer FP predictions of the HPI in those regions. Our alternative statistical approach and the different hemodynamic profile of the patients studied, likely resulted in different sensitivity and specificity numbers, which makes our results less comparable to some previous trials.

To date, performance of the HPI has been externally and clinically validated in five observational studies and three randomized controlled trials (RCTs) [28]. In non-cardiac surgical patients, external validation has been done on invasive [23] and non-invasive [22, 24] arterial waveform data. All these three trials reported sensitivity and specificity > 85%, based on HPI values 5 min prior to the event (in the case of a backward analysis), or based on an HPI value of 85 (in the case of a forward analysis). Furthermore, HPI performance has been evaluated during cardiac surgery, where it performed moderate to good, with sensitivity of 0.62–0.84 and specificity of 0.78–0.84 [27, 29]. These promising results have provided a basis for three randomized clinical trials, to study whether prediction could result in a (clinically relevant) reduction of intra-operative hypotension. The first RCT published compared 25 patients, scheduled for hip arthroplasty, treated with goal-directed HPI-guided hemodynamic therapy to 24 patients receiving routine care. Interventions were initiated at an HPI threshold of 80. Duration of hypotension was significantly reduced in the intervention group [30]. The second RCT was conducted in a heterogeneous surgical population consisting of 68 patients who were assigned to an HPI-guided treatment protocol, which was initiated when a threshold of 85 was exceeded, or to standard care. Compared to the controls, the intervention group spent less time in hypotension [31]. The third RCT conducted in non-cardiac surgery patients, concluded that the HPI did not decrease the amount of hypotension, which might be explained by low hypotension exposure in the control group or insufficient adherence to the treatment protocol [32]. In summary, the HPI has been validated in several clinical settings with moderate to good results, and showed a positive effect on decreasing intraoperative hypotension in two out of three RCTs. However, the ICU is a vastly different setting and patients present with critical illness, compared to relatively healthy patients undergoing surgery. Whether the HPI will decrease hypotension during ICU stay is unknown, but the promising findings of this study add to previous results from earlier trials and provides an entry point for prospective HPI-guided trials in critically ill patients.

Whether prevention of hypotension in ICU patients leads to less morbidity remains unknown, but several retrospective studies reported that hypotension in ICU patients is associated with myocardial injury, AKI, and mortality [10–14]. Not only depth, but also duration is an important determinant of morbidity in these patients [10–12]. Such findings suggest that even short durations of hypotension may impair adequate tissue perfusion and oxygenation and thus may result in organ damage. Although associations between hypotension and complications during or after ICU admission in COVID-19 patients have not been reported, one could hypothesize that this might be the case in this population as well, especially since ICU length of stay in this population is relatively long. In our cohort, 93% of the patients experienced hypotension and the median TWA of hypotension was 0.08 mmHg, which is lower than reported in patients undergoing non-cardiac surgery [31, 33], possibly due to the different factors described before. Nevertheless, hypotension in this cohort was observed in almost all patients, indicating that routine hemodynamic monitoring and treatment could be improved in our institution.

This is a single center, external validation study of the HPI in mechanically ventilated COVID-19 patients. Validation of the HPI should be repeated at various institutions and preferably with larger sample sizes in order to determine the replicability of the current findings. The HPI should also be validated on other (non-COVID-19) ICU patients, to translate the current results to a more heterogeneous ICU population. Furthermore, RCTs with access to HPI data in ICU patients with and without COVID-19, with evaluation of patient outcome would help to determine clinical applicability. A warning on impending hypotension could have a considerable impact on patient care, as predicted hypotensive events could help clinicians to move from a reactive towards a proactive state, thereby minimizing or preventing hypotension exposure. This is especially true during a pandemic, when shortages in staffing may be an issue and machine-learning algorithms can thus be of assistance in monitoring multiple patients at the same time.

### Limitations

There are several limitations of this study that should be addressed. Different definitions apply to hypotension and one definition may not apply to all patients. In a recent international survey among ICU personnel, a MAP < 65 mmHg has been reported most frequently as their currently used definition [34]. Although this definition is also used in our hospital, we may have included patients in this trial where lower or higher blood pressure targets have deliberately been accepted. We did not exclude these patients, which might have influenced our results. Clinical applicability of the HPI is limited to patients requiring a target MAP of 65 mmHg, which is a limitation of the algorithm and not this trial per se, but reduces generalizability of the current findings to ICU settings with MAP targets other than ours.

Furthermore, we obtained measurements with a median duration of 3 [2–3] days per patient, while median length of stay in the ICU was ﻿18 [11–25] days. Although we included patients consecutively, there were no restrictions on when measurements could be obtained during admission. We only refrained from measurements if the expected remaining ventilation period was less than eight h. This led to start of measurements at a median of 6 [3–12] days after ICU admission, possibly affecting the incidence and severity of hypotension.

During measurements, clinical interventions by personnel were not registered, since this was not feasible with multiple simultaneous measurements. In the analyses, we have tried to correct for clinical interventions influencing blood pressure, such as administration of vasopressors, fluids, or application of positional maneuvers. Data exclusions based on rapid blood pressure changes as described in the methods will cover most of these interventions, but we cannot rule out the possibility that some were not excluded, affecting the reported sensitivity, specificity, PPV, and NPV.

## Conclusions

In conclusion, this first external validation of the HPI in ICU patients showed promising results in prediction of impending hypotension. Further studies are warranted to evaluate algorithm performance in a different ICU population, for example, hemodynamically unstable septic patients. We also need to investigate if appropriate clinical intervention following hypotension prediction results in hypotension reduction in ICU patients.

## Data Availability

The datasets used and/or analysed during the current study are available from the corresponding author on reasonable request.

## Code availability

The codes used for analyses are available from the corresponding author on reasonable request.
